# Phenotypic and Genomic Analysis of *Pediococcus acidilactici* MBEL10321 with Potential Probiotic Properties

**DOI:** 10.4014/jmb.2603.03018

**Published:** 2026-06-12

**Authors:** Jeong-Ah Yoon, Min-Kyung Ju, Chan-Haeng Lee, Yoon-Jong Ryu, Young Ju Jin, Myoung-Dong Kim

**Affiliations:** 1 Institute of Fermentation and Brewing, Kangwon National University, Chuncheon, Gangwon 24341, Republic of Korea; 2 Department of Food Biotechnology, Kangwon National University, Chuncheon, Gangwon 24341, Republic of Korea; 3 Department of Otorhinolaryngology-Head and Neck Surgery, Kangwon National University Hospital, Kangwon National University College of Medicine, Chuncheon, Gangwon 24341, Republic of Korea

**Keywords:** *Pediococcus acidilactici*, Antimicrobial activity, Comparative genomic analysis, Antibiotic resistance

## Abstract

This study systematically evaluated the probiotic properties of *Pediococcus acidilactici* MBEL10321, isolated from Nuruk, using both phenotypic and genomic analyses, to assess its functionality and safety. *P. acidilactici* MBEL10321 demonstrated sustained viability under acidic conditions and in the presence of bile salts. The strain also exhibited notable antimicrobial activity against several pathogenic bacteria, including *Staphylococcus aureus*, *Escherichia coli* O157:H7, *Bacillus cereus*, and *Listeria monocytogenes*. Its inhibitory effects exceeded those of the control strains *Lacticaseibacillus rhamnosus* GG and *P. acidilactici* KCTC15064. *P. acidilactici* MBEL10321 did not exhibit hemolytic activity and present safety concerns. Whole-genome analysis revealed the *P. acidilactici* MBEL10321 genome to be 2,044,106 bp, with a G+C content of 42.2%, and to contain 1,980 protein-coding genes. These genomic features were comparable to those of previously reported *P. acidilactici* strains. In addition, no antibiotic-resistance genes, virulence factors, or transferable plasmid replicons were detected. These findings suggest that *P. acidilactici* MBEL10321 is a potential probiotic candidate strain derived from the traditional fermentation starter Nuruk.

## Introduction

Probiotics are live microorganisms that, when administered in adequate amounts, confer health benefits to the host. Their beneficial effects on gut health, immune modulation, and inhibition of pathogenic microorganisms have been widely reported [1]. Lactic acid bacteria (LAB), including the genus *Pediococcus*, have been extensively studied as major probiotic candidates [2], and research on LAB continues to be actively performed, particularly in food science and functional foods.

*Pediococcus* belongs to the LAB and is characterized by a coccoid morphology [3] and has been detected in various fermented foods, such as sausages, Kimchi, and sauerkraut [4-6]. These bacteria are known to stabilize microbial communities during fermentation by producing lactic acid [7]. Moreover, previous studies have reported that *Pediococcus acidilactici* exhibits acid and bile tolerance, as well as antimicrobial activity, suggesting its potential as a probiotic [8, 9].

Nuruk is a traditional Korean fermentation starter produced by natural fermentation of grains using a complex microbial community of fungi, yeasts, and LAB [10]. The manufacturing process of Nuruk involves conditions such as elevated temperature, mildly acidic pH, and limited moisture, which promote microbial competition and selection [11]. Microorganisms surviving this process have been suggested to possess the stress tolerance required for fermentation and food-processing applications [12, 13]. However, despite the recognition of Nuruk as a potential source of functional microbial resources, studies evaluating the probiotic functionality and safety of LAB isolated from Nuruk remain limited.

The probiotic potential of LAB can be assessed through phenotypic evaluations, including acid and bile tolerance, antimicrobial activity, and hemolytic activity. However, these phenotypic traits alone are insufficient to explain the genetic basis of functional properties or to comprehensively assess strain safety [14]. Accordingly, recent studies have increasingly used whole-genome sequencing to evaluate, at the genetic level, the functional potential and safety of probiotic candidate strains with greater precision [15, 16].

In this study, *P. acidilactici* MBEL10321, isolated from Nuruk, was evaluated for its probiotic potential through phenotypic assessments, including acid and bile tolerance, antimicrobial activity, hemolytic activity, and antibiotic susceptibility, as well as whole-genome sequencing.

## Materials and Methods

### Strains

The control strains *Lacticaseibacillus rhamnosus* GG (KCTC5033) and *P. acidilactici* (KCTC15064), used in this study, were obtained from the Korean Collection for Type Cultures (KCTC, Republic of Korea).

### Isolation and Identification of LAB

To isolate LAB from Nuruk, 1 g of the sample was homogenized with 10 mL of sterile distilled water. An aliquot (100 μL) of the homogenized suspension was spread onto MRS agar (MBcell, Republic of Korea) supplemented with 1% bromocresol purple (Daejung, Republic of Korea) and incubated at 37°C for 24 h. A colony forming a yellow halo was selected, designated as MBEL10321, cultivated in MRS broth (MBcell) at 37°C for 24 h, mixed with 15% glycerol, and stored at -80°C.

The 16S rRNA gene was amplified from genomic DNA using the primers 27F (5′-AGAGTTTGATCMTGGCTCAG-3′) and 1492R (5′-TACGGYTACCTTGTTACGACTT-3′). The obtained gene sequences were analyzed for sequence similarity using BLAST at the National Center for Biotechnology Information (NCBI, USA). A phylogenetic tree based on the retrieved sequences was constructed using the neighbor-joining method [17] in MEGA v.12 software, and branch support was assessed by bootstrap analysis with 1,000 replicates.

### Assessment of Acid and Bile Salt Tolerance

Tolerance to acidic and bile conditions was evaluated using a previously described method [18], with minor modifications. The LAB strains were grown to the exponential phase in MRS broth, harvested by centrifugation (9,000 × *g*, 1 min), and washed with sterile distilled water. The washed cells were inoculated into 5 mL of MRS broth adjusted to pH 3.0 with 1 M HCl or into MRS broth supplemented with 0.3% oxgall (Neogen, USA) and incubated at 37°C for 4 h. The cultures were harvested by centrifugation (9,000 × *g*, 1 min) and washed twice with sterile distilled water. The cell suspensions were serially diluted five-fold, and each dilution was spotted onto an MRS agar plate. The plates were incubated at 37°C for 24 h.

Cell viability following acid and bile salt stress was evaluated using Acridine Orange/Propidium Iodide (AOPI) staining coupled with flow cytometric analysis. Briefly, 18 μL of culture was mixed with 2 μL of AOPI viability staining solution (Logos Biosystems, Republic of Korea) and incubated at room temperature for 5 min. Stained cells were analyzed using a CytoFLEX flow cytometer (Beckman Coulter, USA), and 10,000 events were acquired for each sample. Acridine Orange-positive and Propidium Iodide-negative cells were considered viable, whereas Propidium Iodide-positive cells were considered non-viable. Cell viability was expressed as the percentage of viable cells relative to the total cell population.

### Evaluation of Potential Antimicrobial Activity

Antimicrobial activity against pathogenic microorganisms (*Staphylococcus aureus* KCTC1916, *Escherichia coli* O157:H7 KCCM40406, *Bacillus cereus* ATCC14579, *Listeria monocytogenes* KCTC3569, *Pseudomonas fluorescens* KCTC42821, and *Micrococcus luteus* KCTC3063) was evaluated using the disk diffusion method [19]. Each pathogen was grown in tryptic soy broth (MBcell) at 37°C to the exponential phase and then spread onto tryptic soy agar (MBcell). Sterile paper disks were placed on the agar surface, and 10 μL of LAB culture grown to the exponential phase in MRS broth was applied to each disk. The plates were incubated at 30°C or 37°C for 24 h. Antimicrobial activity was assessed by measuring the diameter of the inhibition zones using ImageJ (v1.54p) [20].

### Hemolytic Activity Assay

LAB strains were grown in MRS broth at 37°C to the exponential phase, harvested by centrifugation (9,000 × *g*, 1 min), and washed twice with sterile distilled water. Cell suspensions were adjusted to an optical density at 600 nm (OD_600_) of 0.1, and 20 μL of each suspension was spotted onto blood agar base (MBcell) supplemented with 5% sheep blood defibrinated (MBcell). The plates were incubated at 37°C for 48 h. Hemolytic activity was determined by the appearance of hemolysis zones: the formation of a greenish zone was interpreted as α-hemolysis, a clear zone as β-hemolysis, and the absence of hemolysis as γ-hemolysis [21].

### Antibiotic Susceptibility Assay

Antibiotic susceptibility was evaluated using minimum inhibitory concentration (MIC) test strips (Liofilchem, Italy). MIC values (μg/mL) were determined for eight antibiotics (ampicillin, chloramphenicol, clindamycin, erythromycin, gentamicin, kanamycin, tetracycline, and streptomycin), in accordance with the European Food Safety Authority (EFSA) guidelines. Cell suspensions prepared from the exponential growth phase were adjusted to an OD_600_ of 0.1, spread onto MRS agar plates, and then the MIC test strips were placed on the agar surface. The plates were incubated at 37°C for 24 h. MIC values were determined according to the manufacturer’s instructions.

### Genome Assembly and Annotation

Whole-genome sequencing was performed using both short- and long-read technologies. Short-read data were obtained using the Illumina NovaSeq 6000 system (Illumina, USA), and HiFi long-read sequences were generated on the PacBio Sequel IIe platform (Pacific Biosciences, USA). De novo genome assembly was performed using PacBio HiFi reads with the microbial genome analysis pipeline in SMRT Link (v25.1.0.257715) [22]. The assembled genome was refined by polishing with Inspector (v1.0.1) [23] and then further corrected for errors with Pilon (v1.22) [24] using Illumina short-read data. Genome completeness and contamination were assessed using BUSCO software (v5.1.3) [25]. Gene prediction was performed using Prokka (v1.14.6) [26]. Functional annotation of the predicted protein-coding genes was performed using eggNOG-mapper (v2.1.10) [27] and InterProScan (5.34-73.0) [28] to assign functional categories and protein domains.

### Comparative Genomic Analysis

For comparative genomic analysis, eight *Pediococcus* strains and two *Lacticaseibacillus* strains were selected, and genome sequences registered as reference sequences in the NCBI database were used. Genomic similarity among the strains was evaluated using average nucleotide identity (ANI) values calculated with the OrthoANI Tool (v0.93.1) [29].

To investigate the genetic potential for antimicrobial activity, genes associated with bacteriocin biosynthesis were predicted using BAGEL4 [30], and biosynthetic gene clusters for secondary metabolite production were analyzed using antiSMASH (v6.0) [31].

For genome-based safety assessment, analyses were conducted to identify antibiotic resistance genes (ARGs), virulence factors, plasmid replicons, and prophages. ARGs were detected using the Resistance Gene Identifier (RGI, v6.0.5) based on the Comprehensive Antibiotic Resistance Database (CARD, v4.0.1) [32]. Virulence factors were screened using the Virulence Factor Database (VFDB, 2025 release) [33]. The presence of known plasmid replicons was examined using PlasmidFinder (v2.1) [34], and prophage regions were predicted using PHASTER [35].

### Statistical Analysis

Experiments were performed in triplicate. Data are reported as mean ± SD. Statistical analyses were performed using SPSS Statistics 26 (IBM Corp., USA). Differences among groups were analyzed by one-way analysis of variance (ANOVA) followed by Duncan's multiple range test, with significance defined at *p* < 0.05.

## Results

### Isolation and Identification of LAB

**Fig. 1:**
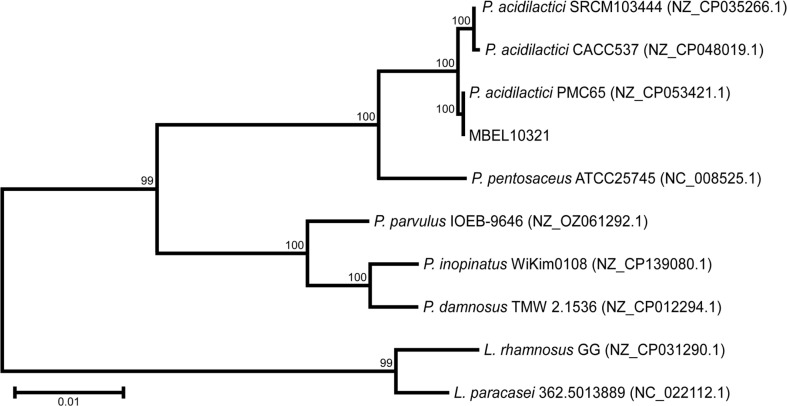
Phylogenetic tree based on 16S rRNA gene sequences from *P. acidilactici* MBEL10321 and other lactic acid bacteria. The tree was constructed using the neighbor-joining method, and bootstrap values (1,000 replicates) are shown at the nodes. The scale bar indicates relative branch lengths.

Among the isolates obtained from Nuruk, strain MBEL10321 formed a yellow halo on MRS agar supplemented with bromocresol purple and was selected for further identification. As shown in Fig. 1, phylogenetic analysis of 16S rRNA gene sequences indicated that strain MBEL10321, isolated from Nuruk, clustered with reference strains of *P. acidilactici*, with strong bootstrap support, and was clearly separated from other LAB. MBEL10321 also exhibited 100% sequence similarity to *P. acidilactici* reference sequences in the NCBI database.

### Assessment of Acid and Bile Salt Tolerance

**Fig. 2:**
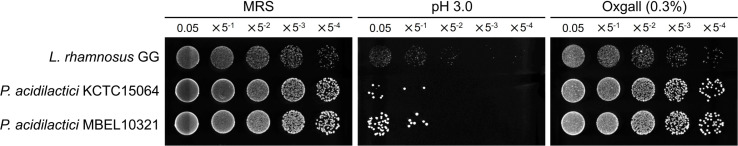
Assessment of acid and bile tolerance of *P. acidilactici* MBEL10321. Cells at the exponential growth phase were exposed to acidic (pH 3.0) or bile salt (0.3% oxgall) conditions at 37°C for 4 h, then spotted onto MRS agar plates to assess growth. The plates were then incubated at 37°C for 24 h. The column headers indicate the initial cell suspension (OD_600_ = 0.05) and subsequent 5-fold serial dilutions.

The growth of *P. acidilactici* MBEL10321 after exposure to acid and bile stress is shown in Fig. 2. At pH 3.0, growth was inhibited in all strains. However, the control strain *L. rhamnosus* GG exhibited relatively higher growth than the *P. acidilactici* strains. Following exposure to 0.3% oxgall, growth inhibition was observed in all strains, but the extent of inhibition was marginal. *P. acidilactici* MBEL10321 showed growth characteristics comparable to those of the control strain *P. acidilactici* KCTC15064 under both acid and bile stress.

Cell viability was quantified by flow cytometry (Table S1). After treatment at pH 3.0, the viability of *P. acidilactici* MBEL10321 was 76.85 ± 3.39%, which was comparable to that of *P. acidilactici* KCTC15064 but lower than that of *L. rhamnosus* GG. Following exposure to 0.3% oxgall, no significant differences in viability were observed among the tested strains. These results were consistent with the acid- and bile-salt-tolerance patterns observed in the spot assay.

### Evaluation of Potential Antimicrobial Activity

The antimicrobial activity of *P. acidilactici* MBEL10321 was assessed by measuring inhibition zones against various pathogens. Strain MBEL10321 produced inhibition zones of 16.00 ± 0.16 mm against *S. aureus* KCTC1916 and 20.46 ± 0.43 mm against *E. coli* O157:H7 KCCM40406, both significantly larger than those observed with the control strain, *P. acidilactici* KCTC15064 (Table 1). Inhibition zones appeared only for MBEL10321 against *B. cereus* ATCC14579 and *L. monocytogenes* KCTC3569. MBEL10321 showed larger inhibition zones against *Pseu. fluorescens* KCTC42821 and *M. luteus* KCTC3063 than the control strains.

### Hemolytic Activity and Antibiotic Susceptibility Assay

**Fig. 3:**
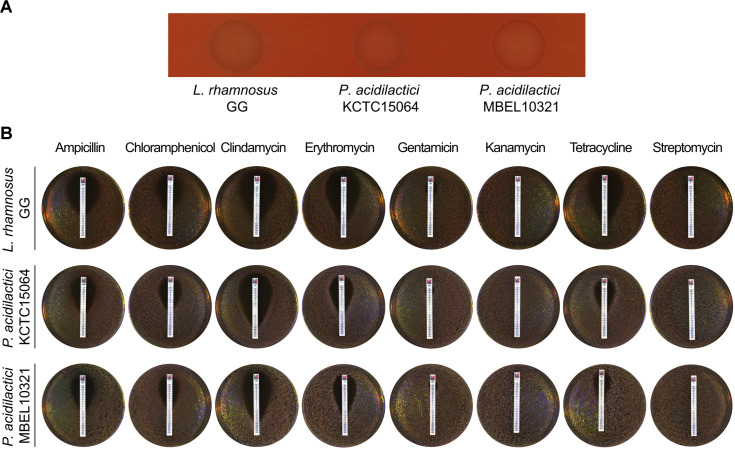
Hemolytic activity (A) and antibiotic susceptibility (B) of *P. acidilactici* MBEL10321. Hemolytic activity was evaluated on blood agar containing 5% (v/v) sheep blood defibrinated, and antibiotic susceptibility was determined using MIC test strips.

*P. acidilactici* MBEL10321 did not exhibit hemolytic activity on blood agar. This finding aligns with those for *P. acidilactici* KCTC15064 and *L. rhamnosus* GG, thereby confirming a γ-hemolytic (non-hemolytic) phenotype (Fig. 3A).

*P. acidilactici* MBEL10321 was susceptible to ampicillin, chloramphenicol, clindamycin, and erythromycin (Fig. 3B, Fig. S1) [36]. In contrast, gentamicin, kanamycin, and streptomycin had MIC values above their respective cut-off thresholds, and similar results were observed in the control strains. Furthermore, MBEL10321 had an MIC value exceeding the established tetracycline threshold. Detailed MIC data for each antibiotic are provided in the supplementary figure (Fig. S1).

### Genome Assembly and Annotation

The total genome size of *P. acidilactici* MBEL10321 was 2,044,106 bp and contained 1,980 protein-coding genes (Table 2). The genome assembly consisted of one complete circular chromosome and one circular plasmid. The chromosome was 1,983,416 bp in length with a G+C content of 42.2% and contained 15 rRNA and 56 tRNA genes, whereas the plasmid was 60,690 bp in length with a G+C content of 39.9% and 73 protein-coding genes. The genome sequence of *P. acidilactici* MBEL10321 has been deposited in the NCBI database under the accession number SRR36748701.

According to the COG functional classification (Table 3), the largest group among the protein-coding genes of *P. acidilactici* MBEL10321 was the “Function unknown (S)” category, comprising 493 genes (25.01% of the total). Carbohydrate transport and metabolism (G) accounted for 182 genes (9.23%), followed by Transcription (K) with 171 genes (8.67%), and Translation, ribosomal structure and biogenesis (J) comprising 159 genes (8.07%). Furthermore, a substantial proportion of genes was classified into the categories Replication, Recombination and Repair (L) and Cell Wall/Membrane/Envelope Biogenesis (M). In contrast, no genes were found in the categories of RNA processing and modification (A), Chromatin structure and dynamics (B), General function prediction only (R), Nuclear structure (Y), Extracellular structures (W), or Cytoskeleton (Z). Additionally, only a few genes were found in the Cell motility (N) category.

### Comparative Genomic Analysis

The genome size and G+C content of MBEL10321 were comparable to those of reference *P. acidilactici* strains (PMC65, CACC537, and SRCM103444) (Table 2). On the other hand, MBEL10321 harbored a plasmid, resulting in a marginal increase in the number of protein-coding genes. Other *Pediococcus* species differed from MBEL10321 in genome size, G+C content, and plasmid content. *L. rhamnosus* GG and *L. paracasei* 362.5013889 had larger genomes, more protein-coding genes, and higher G+C contents (about 46.5%) than *Pediococcus* strains, indicating clear genomic differences.

**Fig. 4:**
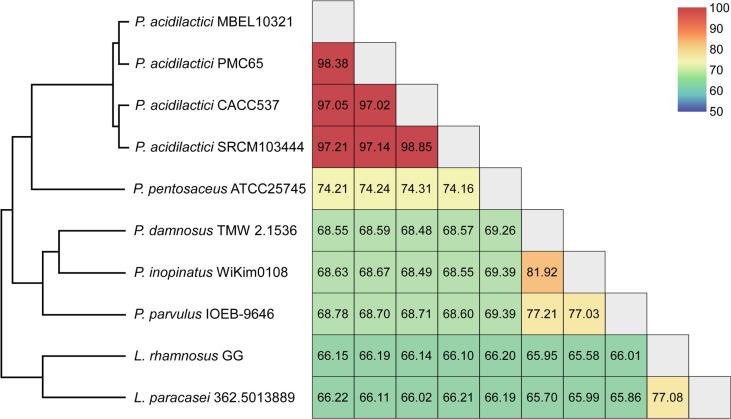
Average nucleotide identity (ANI) heatmap of the genomes of *P. acidilactici* MBEL10321 and other lactic acid bacteria. ANI values were calculated using the OrthoANI Tool (v0.93.1). The color scale represents pairwise ANI values. Hierarchical clustering was performed using the ANI distance.

ANI analysis was conducted to assess the genomic similarity of *P. acidilactici* MBEL10321 (Fig. 4). MBEL10321 had ANI values above 97% relative to those of reference strains of *P. acidilactici* and clustered with them, providing genomic evidence that MBEL10321 is a *P. acidilactici* strain. On the other hand, MBEL10321 showed ANI values of 68–74% relative to those of other *Pediococcus* species, indicating limited genomic similarity. MBEL10321 was distinct from strains classified in the genus *Lacticaseibacillus*.

In *P. acidilactici* MBEL10321, a single terpene precursor–type biosynthetic gene cluster was predicted, which was also reported in *P. acidilactici* PMC65 (Table 4, Table S2). In contrast, *P. acidilactici* CACC537 and SRCM103444 harbored two biosynthetic gene clusters: a terpene precursor cluster and a type III polyketide synthase (T3PKS) cluster. Other LAB strains were predicted to possess 2–4 secondary metabolite–related clusters. Bacteriocin gene clusters were not detected in *P. acidilactici* strains, including MBEL10321 (Tables 4 and S3). However, other LAB strains were predicted to harbor various bacteriocin gene clusters, including pediocin, plantaricin, carnocin, and thermophilin.

ARGs, virulence factors, plasmids, and prophages were analyzed in *P. acidilactici* MBEL10321 and LAB strains for safety assessment (Table 5). Perfect-hit ARGs were not detected in any of the analyzed strains. Except for *L. rhamnosus* GG, one to four strict hits were detected in the analyzed strains; however, these hits exhibited relatively low sequence identities ranging from 31.15% to 49.02% (Table S4). Furthermore, aminoglycoside resistance genes associated with resistance to gentamicin, kanamycin, or streptomycin were not detected. Virulence factors were also absent from all analyzed strains. Replicons were not predicted in *P. acidilactici* strains, including MBEL10321, whereas a single plasmid replicon was identified only in *P. damnosus* TMW 2.1536 and *L. paracasei* 362.5013889. Two intact and two incomplete prophage regions were predicted in *P. acidilactici* MBEL10321. Other LAB strains also harbored intact, questionable, and incomplete prophage regions, with differences in the number and distribution of intact prophages across strains.

## Discussion

In this study, the probiotic potential of *P. acidilactici* MBEL10321, isolated from Nuruk, was evaluated through phenotypic and genomic assessments of functionality and safety. Growth of *P. acidilactici* MBEL10321 was inhibited under acidic conditions, but complete inactivation was not observed. Only mild growth inhibition was observed at 0.3% bile salt, suggesting that MBEL10321 may survive under conditions simulating the gastrointestinal environment, consistent with previous reports on *P. acidilactici* isolated from fermented grains [37].

Antimicrobial activity is an important indicator of the probiotic potential of candidate strains. *P. acidilactici* MBEL10321 exhibited antimicrobial activity against a range of pathogenic strains, and its inhibitory effects were generally higher than those of the control strains *L. rhamnosus* GG and *P. acidilactici* KCTC15064. The antibacterial activity of *P. acidilactici* has been observed in strains from various fermented foods—including meat [22] and grain-based products [38]—with inhibition reported against pathogens such as *S. aureus*, *L. monocytogenes*, and *E. coli*. These antibacterial properties reportedly vary across strains [39]. The relatively strong antibacterial activity observed for MBEL10321 indicates that it is a functionally distinct strain within the same species, with potential competitiveness in intestinal or fermentation environments.

*P. acidilactici* MBEL10321 exhibited a γ-hemolytic (non-hemolytic) phenotype, indicating the absence of pathogenic potential associated with erythrocyte lysis. In antibiotic susceptibility assays, *P. acidilactici* MBEL10321 exhibited MIC values exceeding the EFSA cut-off thresholds for gentamicin, kanamycin, streptomycin, and tetracycline. Previous studies [40, 41] reported elevated MICs for gentamicin, kanamycin, streptomycin, and tetracycline in *P. acidilactici* strains. According to EFSA guidelines [36], phenotypic antibiotic resistance observed in LAB may reflect intrinsic characteristics at the species or genus level. It should not be interpreted as a safety concern based solely on phenotypic results. Therefore, to accurately determine whether resistance is acquired, analysis of antibiotic resistance genes should be conducted in parallel.

The genome size, G+C content, and COG functional category distribution of *P. acidilactici* MBEL10321 were comparable to those previously reported for *P. acidilactici* strains [42]. These findings suggest that MBEL10321 exhibits genomic features typical of the species.

Bacteriocin biosynthetic gene clusters were not identified in the genome of *P. acidilactici* MBEL10321, as in reference *P. acidilactici* strains. Accordingly, the antimicrobial activity observed for MBEL10321 is likely attributable to nonspecific factors, such as organic acid production or nutrient competition, rather than to bacteriocin-mediated mechanisms. This is supported by previous findings showing that the antimicrobial effects of *P. acidilactici*-derived cell-free supernatants are primarily due to organic acid accumulation rather than to proteinaceous bacteriocins [43].

Genome-based safety assessment did not detect ARGs, virulence factors, and transferable plasmid replicons in the genome of *P. acidilactici* MBEL10321. Although several prophage regions were identified, these elements were also commonly observed in the reference LAB strains and were not directly associated with antibiotic resistance or virulence genes. Accordingly, MBEL10321 does not appear to harbor genomic risk factors for ARG dissemination or pathogenicity, and the antimicrobial activity observed in phenotypic assays is likely attributable to the strain's intrinsic metabolic and physiological properties.

Although *P. acidilactici* is not generally regarded as a dominant autochthonous member of the human gastrointestinal microbiota, several strains have been investigated as potential probiotic candidates [8, 9, 42]. In the present study, the adhesion ability of MBEL10321 was not experimentally evaluated. However, genome analysis identified several putative adhesion-related proteins, including a MucBP (mucin-binding protein) domain-containing protein, sortase A, and a collagen-binding domain-containing protein. These proteins have been reported to contribute to host surface interactions in lactic acid bacteria [44], suggesting the potential of MBEL10321 to interact with the gastrointestinal environment. Nevertheless, the presence of these adhesion-related genes alone does not confirm adhesion ability, and further functional validation is required.

In summary, *P. acidilactici* MBEL10321 exhibited tolerance to acidic and bile conditions and showed antimicrobial activity. It did not raise safety concerns based on the absence of hemolytic activity, antibiotic susceptibility assessments, and genome-based analyses. Therefore, *P. acidilactici* MBEL10321 is considered a potential probiotic candidate strain derived from the traditional fermentation starter Nuruk. Although MBEL10321 exhibited probiotic characteristics generally comparable to those reported for established probiotic strains, the present study is significant in that it demonstrates the probiotic potential of a microorganism isolated from the traditional Korean fermentation starter, Nuruk, thereby highlighting the value of Nuruk-derived microbial resources as probiotic candidates. In addition, integrating phenotypic characterization with genome-based safety assessment enabled a more comprehensive evaluation of the strain's suitability for probiotic applications.

## Supplemental Materials

Supplementary data for this paper are available on-line only at http://jmb.or.kr.



## Figures and Tables

**Table 1:** Antibacterial activity of *P. acidilactici* MBEL10321 against pathogenic bacteria^a)^.

Pathogen	Inhibition zone (diameter, mm)		
	*L. rhamnosus* GG	*P. acidilactici* KCTC15064	*P. acidilactici* MBEL10321
*Staphylococcus aureus* KCTC1916	ND^b)^	12.90 ± 0.25^a^	16.00 ± 0.16^b^
*Escherichia coli* O157:H7 KCCM40406	ND	6.23 ± 1.43^a^	20.46 ± 0.43^b^
*Bacillus cereus* ATCC14579	ND	ND	22.01 ± 0.39
*Listeria monocytogenes* KCTC3569	ND	ND	10.78 ± 0.22
*Pseudomonas fluorescens* KCTC42821	8.83 ± 0.07^b^	7.90 ± 0.02^a^	12.56 ± 0.10^c^
*Micrococcus luteus* KCTC3063	9.10 ± 0.28^a^	10.83 ± 0.05^b^	14.00 ± 0.06^c^

^a)^ Different superscripts indicate statistically significant differences within the same row (*p* < 0.05)

^b)^ ND, not detected

**Table 2:** Comparison of genomic features of *P. acidilactici* MBEL10321 and other lactic acid bacteria.

Strain	Total genome size (bp)	Protein-coding gene	Chromosome				Plasmid (Size, bp)	GenBank Accession No.
			Length (bp)	G+C (%)	rRNA	tRNA		
*P. acidilactici* MBEL10321	2,044,106	1,980	1,983,416	42.2	15	56	1 (60,690)	-
*P. acidilactici* PMC65	2,044,083	1,915	2,044,083	42.0	15	57	-	GCF_013127755.1
*P. acidilactici* CACC537	2,035,984	1,864	2,035,984	42.0	15	56	-	GCF_010092385.1
*P. acidilactici* SRCM103444	1,970,727	1,806	1,970,727	42.0	15	56	-	GCF_004103635.1
*P. pentosaceus* ATCC25745	1,832,387	1,735	1,832,387	37.5	15	55	-	GCF_000014505.1
*P. damnosus* TMW 2.1536	2,173,661	1,968	2,125,430	38.0	12	62	2 (7,678, 40,553)	GCF_001611155.1
*P. inopinatus* WiKim0108	2,090,142	1,943	2,022,758	38.5	12	62	2 (16,138, 51,246)	GCF_034049535.1
*P. parvulus* IOEB-9646	2,163,309	2,065	2,090,911	38.5	12	62	2 (27,848, 44,550)	GCF_964063155.1
*L. rhamnosus* GG	3,010,116	2,699	3,010,116	46.5	15	57	-	GCF_003353455.1
*L. paracasei* 362.5013889	3,025,352	2,808	2,939,026	46.5	15	59	2 (24,207, 62,119)	GCF_000155515.2

**Table 3:** Distribution of protein-coding genes in *P. acidilactici* MBEL10321 according to COG functional categories.

COG category	Description	Count	Ratio (%)
A	RNA processing and modification	0	0.00
B	Chromatin structure and dynamics	0	0.00
C	Energy production and conversion	72	3.65
D	Cell cycle control, cell division, chromosome partitioning	27	1.37
E	Amino acid transport and metabolism	98	4.97
F	Nucleotide transport and metabolism	112	5.68
G	Carbohydrate transport and metabolism	182	9.23
H	Coenzyme transport and metabolism	54	2.74
I	Lipid transport and metabolism	50	2.54
J	Translation, ribosomal structure and biogenesis	159	8.07
K	Transcription	171	8.67
L	Replication, recombination and repair	149	7.56
M	Cell wall/membrane/envelope biogenesis	118	5.99
N	Cell motility	8	0.41
O	Posttranslational modification, protein turnover, and chaperones	39	1.98
P	Inorganic ion transport and metabolism	79	4.01
Q	Secondary metabolites biosynthesis, transport and catabolism	23	1.17
R	General function prediction only	0	0.00
S	Function unknown	493	25.01
T	Signal transduction mechanisms	43	2.18
U	Intracellular trafficking, secretion, and vesicular transport	49	2.49
V	Defense mechanisms	45	2.28
W	Extracellular structures	0	0.00
Y	Nuclear structure	0	0.00
Z	Cytoskeleton	0	0.00

**Table 4:** *In silico* prediction of putative antimicrobial-associated gene clusters in *P. acidilactici* MBEL10321 and other lactic acid bacteria^a)^.

Strain	antiSMASH	BAGEL4
*P. acidilactici* MBEL10321	1	ND^b)^
*P. acidilactici* PMC65	1	ND
*P. acidilactici* CACC537	2	ND
*P. acidilactici* SRCM103444	2	ND
*P. pentosaceus* ATCC25745	3	2
*P. damnosus* TMW 2.1536	2	1
*P. inopinatus* WiKim0108	2	1
*P. parvulus* IOEB-9646	3	1
*L. rhamnosus* GG	3	1
*L. paracasei* 362.5013889	4	2

^a)^ Predictions were performed using antiSMASH and BAGEL4, and the numbers indicate the counts of predicted gene clusters

^b)^ ND, not detected

**Table 5:** *In silico* prediction of safety-related genetic elements in *P. acidilactici* MBEL10321 and other lactic acid bacteria^a)^.

Strain	Antibiotic resistance gene			Virulence factor	Plasmid replicon	Prophage		
	Perfect hit	Strict hit	Loose hit			Intact	Questionable	Incomplete
*P. acidilactici* MBEL10321	ND^b)^	3	ND	ND	ND	2	ND	2
*P. acidilactici* PMC65	ND	3	ND	ND	ND	1	1	1
*P. acidilactici* CACC537	ND	4	ND	ND	ND	1	ND	
*P. acidilactici* SRCM103444	ND	3	ND	ND	ND	ND	ND	5
*P. pentosaceus* ATCC25745	ND	2	ND	ND	ND	2	ND	1
*P. damnosus* TMW 2.1536	ND	2	ND	ND	1	ND	ND	1
*P. inopinatus* WiKim0108	ND	2	ND	ND	ND	1	1	1
*P. parvulus* IOEB-9646	ND	1	ND	ND	ND	1	2	1
*L. rhamnosus* GG	ND	ND	ND	ND	ND	ND	1	3
*L. paracasei* 362.5013889	ND	1	ND	ND	1	1	2	2

^a)^ Predictions were performed using RGI (CARD), VFDB, PlasmidFinder, and PHASTER, and the numbers indicate the counts of detected elements

^b)^ ND, not detected
