# Supplementary material for: Phenotypic and Genomic Analysis of Pediococcus acidilactici MBEL10321 with Potential Probiotic Properties
Source: J Microbiol Biotechnol. 2026 Jun 12;36:e2603018. doi: 10.4014/jmb.2603.03018 (PMC13334079; doi:10.4014/jmb.2603.03018)
Supplement: Supplementary file 1 [file jmb-36-e2603018-supple.pdf]

Supporting Information

Table S1. Cell viability of *P. acidilactici* MBEL10321 following exposure to acidic (pH 3.0) and bile salt (0.3% oxgall) conditions.

**Condition**	**Cell viability (%)**		
	***L. rhamnosus* GG**	***P. acidilactici* KCTC15064**	***P. acidilactici* MBEL10321**
pH3.0	88.44 ± 2.15[b]	77.83 ± 3.06[a]	76.85 ± 3.39[a]
0.3% oxgall	74.66 ± 3.00[a]	75.96 ± 2.04[a]	76.85 ± 3.40[c]

[a)] Different superscripts indicate statistically significant differences within the same row ($p < 0.05$)

Table S2. Biosynthetic gene clusters predicted by antiSMASH in *P. acidilactici* MBEL10321 and other lactic acid bacteria.

Strain	Cluster type	Genomic location
P. acidilactici MBEL10321	Terpene precursor	832,236 – 853,120
P. acidilactici PMC65	Terpene precursor	855,426 – 876,310
P. acidilactici CACC537	T3PKS[a]	1,824,426 – 1,865,595
	Terpene precursor	1,881,493 – 1,902,377
P. acidilactici SRCM103444	Terpene precursor	808,734 – 829,618
	T3PKS	845,140 – 886,309
P. pentosaceus ATCC25745	RiPP-like[b]	85,015 – 100,106
	Terpene precursor	815,674 – 836,564
	T3PKS	850,137 – 891,303
P. damnosus TMW 2.1536	Terpene precursor	1,862,774 – 1,883,676
	T3PKS	2,102,080 – 2,125,430
P. inopinatus WiKim0108	Terpene precursor	929,611 – 970,783
	T3PKS	1,154,381 – 1,175,283
P. parvulus IOEB-9646	RRE-element containing cluster	30,460 – 51,563
	T3PKS	1,044,917 – 1,086,086
	Terpene precursor	1,267,226 – 1,288,131
L. rhamnosus GG	Terpene precursor	1,696,223 – 1,717,074
	T3PKS	1,846,928 – 1,888,100
	RiPP-like	2,456,480 – 2,468,672
L. paracasei 362.5013889	Terpene	1,194,224 – 1,214,847
	Terpene precursor	1,663,199 – 1,684,050
	RiPP-like	2,373,758 – 2,396,867
	RiPP-like	2,700,226 – 2,710,480

[a] T3PKS, type III polyketide synthase

[b] RiPP-like, other unspecified ribosomally synthesized and post-translationally modified peptide product

Table S3. Bacteriocin gene clusters predicted by BAGEL4 in *P. acidilactici* MBEL10321 and other lactic acid bacteria.

Strain	Predicted bacteriocin	Genomic location
P. acidilactici MBEL10321	ND[a)]	-
P. acidilactici PMC65	ND	-
P. acidilactici CACC537	ND	-
P. acidilactici SRCM103444	ND	-
P. pentosaceus ATCC25745	Penocin_A	80,017 – 100,194
	Enterolysin_A	800,894 – 821,227
P. damnosus TMW 2.1536	Pediocin	874,810 - 895107
P. inopinatus WiKim0108	Plantaricin_N	778,261 - 798414
P. parvulus IOEB-9646	Carnocin_CP52	2,041,931 – 2,062,159
L. rhamnosus GG	Carnocin_CP52	2,459,651 – 2,485,540
L. paracasei 362.5013889	ThermophilinA	2,695,230 – 2,715,461
	LSEI_2386	2,371,419 – 2,399,990

[a)] ND, not detected

Table S4. Antimicrobial resistance gene hits classified as 'strict hits' by CARD-RGI in *P. acidilactici* MBEL10321 and other lactic acid bacteria.

Strain	ARO[a)] Term	Drug class	Resistance mechanism	Identity (%)	Coverage (%)
P. acidilactici MBEL10321	sdrM	Fluoroquinolone antibiotic, disinfecting agents and antiseptics	Antibiotic efflux	34.81	109.40
	vanT (vanG cluster)	Glycopeptide antibiotic	Antibiotic target alteration	31.15	52.67
	qacG	Disinfecting agents and antiseptics	Antibiotic efflux	48.11	99.07
P. acidilactici PMC65	sdrM	Fluoroquinolone antibiotic; disinfecting agents and antiseptics	Antibiotic efflux	34.81	109.40
	vanT (vanG cluster)	Glycopeptide antibiotic	Antibiotic target alteration	32.07	52.67
	qacG	Disinfecting agents and antiseptics	Antibiotic efflux	48.11	99.07
P. acidilactici CACC537	sdrM	Fluoroquinolone antibiotic; disinfecting agents and antiseptics	Antibiotic efflux	34.81	109.40
	qacG	Disinfecting agents and antiseptics	Antibiotic efflux	48.11	99.07
	vanT (vanG cluster)	Glycopeptide antibiotic	Antibiotic target alteration	31.69	52.67
	qacJ	Disinfecting agents and antiseptics	Antibiotic efflux	43.81	99.07
P. acidilactici SRCM103444	qacG	Disinfecting agents and antiseptics	Antibiotic efflux	48.11	99.07
	vanT (vanG cluster)	Glycopeptide antibiotic	Antibiotic target alteration	31.69	52.67
	sdrM	Fluoroquinolone antibiotic; disinfecting agents and antiseptics	Antibiotic efflux	34.81	109.40
P. pentosaceus ATCC25745	qacJ	Disinfecting agents and antiseptics	Antibiotic efflux	48.57	100.93
	vanT (vanG cluster)	Glycopeptide antibiotic	Antibiotic target alteration	32.70	52.53
P. damnosus TMW 2.1536	qacJ	Disinfecting agents and antiseptics	Antibiotic efflux	49.02	98.13
	vanT (vanG cluster)	Glycopeptide antibiotic	Antibiotic target alteration	32.97	52.67

P. inopinatus WiKim0108	vanT (vanG cluster)	Glycopeptide antibiotic	Antibiotic target alteration	32.26	52.67
	qacJ	Disinfecting agents and antiseptics	Antibiotic efflux	48.04	98.13
P. parvulus IOEB-9646	vanT (vanG cluster)	Glycopeptide antibiotic	Antibiotic target alteration	31.61	52.67
L. rhamnosus GG	ND[b)]	ND	ND	ND	ND
L. paracasei 362.5013889	qacJ	Disinfecting agents and antiseptics	Antibiotic efflux	38.24	99.07

[a)] ARO, antibiotic resistance ontology

[b)] ND, not detected

Supplementary figure legends

Fig. S1. Minimum inhibitory concentrations (MICs) of antibiotics for *L. rhamnosus* GG (A) and *P. acidilactici* KCTC15064 and MBEL10321 (B). MICs for *L. rhamnosus* GG (white bars), *P. acidilactici* KCTC15064 (light gray), and MBEL10321 (dark gray) were compared with EFSA cut-off values (black bars). Error bars represent the standard deviation across three independent experiments.

Fig. S1

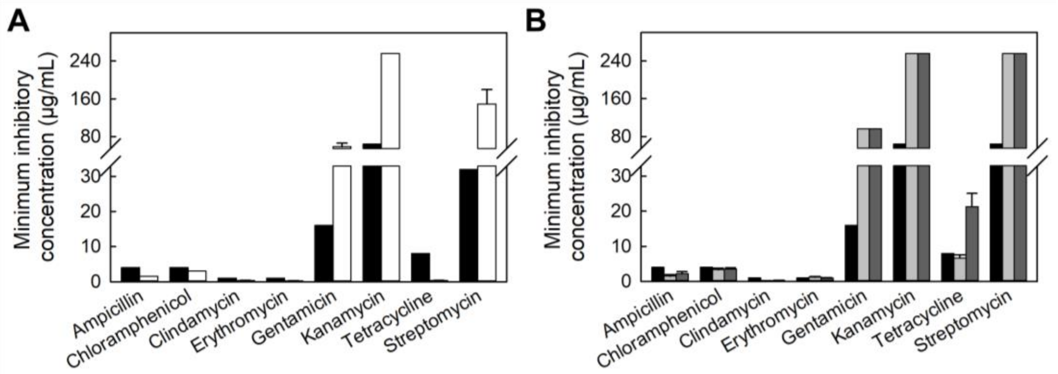
A
B
Minimum inhibitory concentration (µg/mL)
240
160
80
30
20
10
0
Ampicillin
Chloramphenicol
Clindamycin
Erythromycin
Gentamicin
Kanamycin
Tetracycline
Streptomycin
